# Roles of adenosine A_1_ receptors in the regulation of SFK activity in the rat forebrain

**DOI:** 10.1002/brb3.2254

**Published:** 2021-06-22

**Authors:** Li‐Min Mao, John Q. Wang

**Affiliations:** ^1^ Department of Biomedical Sciences School of Medicine University of Missouri‐Kansas City Kansas City Missouri USA; ^2^ Department of Anesthesiology School of Medicine University of Missouri‐Kansas City Kansas City Missouri USA

**Keywords:** RRID:SCR_002798, caudate putamen, DPCPX, Fyn, nucleus accumbens, prefrontal cortex, RRID:AB_10013641, RRID:AB_2106047, RRID:AB_2106058, RRID:AB_476693, RRID:AB_627642, RRID:AB_631528, RRID:RGD_2312511, Src

## Abstract

Adenosine A_1_ receptors are widely expressed in the mammalian brain. Through interacting with G_αi/o_‐coupled A_1_ receptors, the neuromodulator adenosine modulates a variety of cellular and synaptic activities. To determine the linkage from A_1_ receptors to a key intracellular signaling pathway, we investigated the impact of blocking A_1_ receptors on a subfamily of nonreceptor tyrosine kinases, that is, the Src family kinase (SFK), in different rat brain regions in vivo. We found that pharmacological blockade of A_1_ receptors by a single systemic injection of the A_1_ selective antagonist 8‐cyclopentyl‐1,3‐dipropylxanthine (DPCPX) induced an increase in autophosphorylation of SFKs at a consensus activation site, tyrosine 416 (Y416), in the two subdivisions of the striatum, the caudate putamen and nucleus accumbens. DPCPX also increased SFK Y416 phosphorylation in the medial prefrontal cortex (mPFC) but not the hippocampus. The DPCPX‐induced Y416 phosphorylation was time dependent and reversible. In immunopurified Fyn and Src proteins from the striatum, DPCPX elevated SFK Y416 phosphorylation and tyrosine kinase activity in Fyn but not in Src proteins. In the mPFC, DPCPX enhanced Y416 phosphorylation and tyrosine kinase activity in both Fyn and Src immunoprecipitates. DPCPX had no effect on expression of total Fyn and Src proteins in the striatum, mPFC, and hippocampus. These results demonstrate a tonic inhibitory linkage from A_1_ receptors to SFKs in the striatum and mPFC. Blocking this inhibitory tone could significantly enhance constitutive SFK Y416 phosphorylation in the rat brain in a region‐ and time‐dependent manner.

## Graphical abstract

Pharmacological blockade of adenosine A1 receptors enhanced phosphorylation and enzymatic activity of the Src family kinases such as Fyn and/or Src in the adult rat brain in a region‐ and time‐dependent manner. This supports an existence of a tonic inhibitory linkage from A1 receptors to a key and Fyn/Src‐dependent intracellular signaling pathway in neurons in vivo under normal conditions.

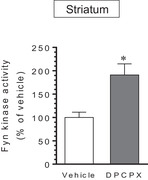

## 1 SIGNIFICANCE

Adenosine is an essential modulator in the central nervous system. Through interactions with G_αi/o_‐coupled A_1_ receptors, adenosine vigorously modulates a variety of cellular and synaptic activities. In this study, we found that pharmacological blockade of A_1_ receptors enhanced phosphorylation and enzymatic activity of the Src family kinases such as Fyn and/or Src in the adult rat brain in a region‐ and time‐dependent manner. This supports an existence of a tonic inhibitory linkage from A_1_ receptors to a key and Fyn/Src‐dependent intracellular signaling pathway in the rat forebrain in vivo under normal conditions.

Adenosine receptors are a class of purinergic G protein‐coupled receptors and are activated by an endogenous neuromodulator ligand, that is, a ubiquitous purine nucleoside adenosine (Fredholm et al., 2001; Sheth et al., 2014). Four subtypes of adenosine receptors (A_1_, A_2A_, A_2B_, and A_3_) have been identified to date (Fredholm, 2011). Each of these receptors is encoded by a separate gene and plays either different or some overlapping roles. At the G protein level, adenosine A_1_ and A_3_ receptors are coupled to G_αi/o_ proteins, whereas adenosine A_2A_ and A_2B_ receptors are coupled to G_αs/olf_ proteins. As a result, activation of A_1_ and A_3_ receptors inhibits adenylyl cyclase and thereby reduces cytoplasmic cAMP levels and protein kinase A (PKA) activity, whereas activation of A_2A_ and A_2B_ receptors produces the opposite effect (Fredholm et al., 2001; Kull et al., 2000; Sheth et al., 2014). In addition to the canonical cAMP pathway, adenosine receptors may trigger other signaling pathways to achieve their regulatory roles in a variety of cellular and synaptic activities (Borea et al., 2016; Fredholm et al., 2001; Schulte & Fredholm, 2003; Sheth et al., 2014).

Among four subtypes of adenosine receptors, A_1_ and A_2A_ receptors are principally expressed in the brain (Fredholm et al., 2005). Especially, A_1_ receptors, the most conserved subtype among species (Fredholm et al., 2000), are distributed throughout the body with the highest level in the brain. A wide range of brain regions express A_1_ receptors, including the cortex, hippocampus, cerebellum and striatum (Alexander & Reddington, 1989; J. F. Chen et al., 2013; Mahan et al., 1991), indicating their roles in the large range of physiological events (Kashfi et al., 2017; Sawynok, 2016; Varani et al., 2017,2016). Within the striatum, A_1_ receptors are expressed in a specific subpopulation of medium spiny projection neurons, that is, striatonigral neurons (Fuxe et al., 2007; 2010) in which dopamine D_1_ receptors are co‐expressed (Aubert et al., 2000; Bertran‐Gonzalez et al., 2010; Gerfen et al., 1990). In fact, behavioral and biochemical studies revealed a postsynaptic A_1_‐D_1_ receptor‐receptor interaction in the rat striatum (Ferre et al., 1994). The two receptors (A_1_ and D_1_) coimmunoprecipitated in co‐transfected fibroblast cells (Gines et al., 2000) and nucleus accumbens (NAc) neurons (Toda et al., 2003). Given that D_1_ receptors are coupled to G_αs/olf_ proteins and thus stimulate the cAMP pathway when activated (Neve et al., 2004), A_1_ receptors are thought to antagonistically modulate striatal D_1_ signaling and fine‐tune the outflow of the D_1_‐mediated direct pathway in the basal ganglia (reviewed in Fuxe et al., 2007, 2010).

The Src family kinase (SFK) is a family of nonreceptor tyrosine kinases (Neet & Hunter, 1996). Five out of nine SFK members (Src, Fyn, Lyn, Yes, and Lck) are expressed in the brain (S. Chen et al., 1996; Omri et al., 1996; Kalia et al., 2004). Among them, Src and Fyn are key members of SFKs and are enriched at synaptic sites (Ohnishi et al., 2011). Accumulating evidence consistently shows that Src and Fyn, by phosphorylating specific tyrosine sites on their substrates, regulate various neural and synaptic activities (Kalia et al., 2004; Ohnishi et al., 2011; Schenone et al., 2011). Meanwhile, Src and Fyn are among postreceptor kinases that are sensitive to changing cellular and synaptic input. By upregulating phosphorylation of Src and Fyn at an autophosphorylation site, tyrosine 416 (Y416), Src and Fyn kinase activity is enhanced drastically (Roskoski, 2005; Okada, 2012). However, whether Src and/or Fyn in striatal neurons are sensitive to A_1_ receptor signaling remains elusive to date.

We thus initiated the present study to investigate the possible linkage between A_1_ receptors and SFKs. To this end, we examined and characterized the effect of pharmacological blockade of A_1_ receptors by a systemic injection of an A_1_ selective antagonist on SFK Y416 phosphorylation, that is, activation of SFKs, in the two subdivisions of the rat striatum, the caudate putamen (CPu) and NAc. Other brain regions were also surveyed, which include the medial prefrontal cortex (mPFC) and hippocampus.

## 2 MATERIALS AND METHODS

### 2.1 Animals

The study was not pre‐registered. Adult male Wistar rats (8‐12 weeks of age, 280–380 g, RRID:RGD_2312511; catalog #: 2312511) that were purchased from Charles River (New York, NY) were kept at a facility (12‐h light/12‐h dark cycle, 23°C) with water and food available ad libitum. Animals were housed two rats per cage and were used 5–6 days after habituation. The animal use and care protocol was approved by the Institutional Animal Care and Use Committee of University of Missouri‐Kansas City (protocol #: 1006).

### 2.2 Experimental arrangements

We randomly divided rats into different groups using a computer‐generated randomization table (GraphPad software/QuickCalcs, La Jolla, CA) and determined sample size by the sample size calculation (alpha = 0.05, beta = 0.2; 80% power). Sample size was not different between the beginning and end of the experiments. Inclusion/exclusion criteria were based on the health state of animals. We used an intraperitoneal (i.p.) injection to administer drugs. Drugs were calculated as the salt for their doses and were injected in a volume of 1 ml/kg. Age‐matched rats received a vehicle injection and served as controls. We first examined effects of the A_1_ antagonist 8‐cyclopentyl‐1,3‐dipropylxanthine (DPCPX) on SFK Y416 phosphorylation in a total of three groups (*n *= 4 per group). DPCPX is selective for A_1_ receptors as it was approximately 1000‐fold more potent at A_1_ than A_2A_ receptors in the striatum (Fredholm & Lindstrom, 1999). After an i.p. injection of vehicle or DPCPX at either 0.25 or 2.5 mg/kg, rats were sacrificed 20 min after drug injection for subsequent Western blot analysis of changes in Y416 phosphorylation in four different brain regions (CPu, NAc, mPFC, and hippocampus). Second, we carried out a time course study. Rats were sacrificed at different time points (20 min, 1 h, and 3 h) after an i.p. injection of vehicle or DPCPX (2.5 mg/kg). Two groups of rats were used for each time point (*n* = 4–5 per group). Changes in Y416 phosphorylation were analyzed in the CPu, NAc, and mPFC. Third, we attempted to identify the members of SFKs that were regulated by A_1_ receptor signaling in two groups of rats (*n* = 4 per group). To this end, we analyzed changes in Y416 phosphorylation in immunopurified Fyn and Src proteins from the striatum and mPFC after vehicle or DPCPX administration (2.5 mg/kg, i.p.; 20–30 min). Finally, tyrosine kinase activity of immunopurified Fyn and Src was assayed in two groups of rats (*n* = 5 per group). Changes in Fyn and Src kinase activity in response to vehicle or DPCPX (2.5 mg/kg, i.p.; 20–30 min) were monitored in the rat striatum and mPFC. Doses of DPCPX (0.25 and 2.5 mg/kg) were chosen based on previous findings that an i.p. injection of DPCPX at 3 mg/kg blocked the anxiolytic‐like effect induced by the A_1_ selective agonist and positive allosteric modulator (Prediger et al., 2006; Vincenzi et al., 2016).

### 2.3 Western blot

Rats were anesthetized with sodium pentobarbital (65 mg/kg, i.p.) and decapitated. Brains were removed and coronal slices were cut. The brain regions of interest (CPu, NAc, and hippocampus) were dissected. In addition, the mPFC was dissected, which includes the anterior cingulate, prelimbic, and infralimbic cortex (Heidbreder & Groenewegen, 2003). Brain tissue was homogenized in radioimmunoprecipitation assay (RIPA) buffer containing 20 mM Tris‐HCl, pH 7.5, 150 mM NaCl, 1 mM Na_2_EDTA, 1 mM EGTA, 1% NP‐40, 1% sodium deoxycholate, 2.5 mM sodium pyrophosphate, 1 mM β‐glycerophosphate, 1 mM Na_3_VO_4_, and 1 μg/ml leupeptin (Cell Signaling Technology, Danvers, MA). Samples were centrifuged at 800 g (10 min) and the supernatant was collected. After protein concentrations were determined, samples were used for immunoblots. Western blot was performed as described previously (Mao et al., 2013; Mao & Wang, 2015). Briefly, proteins were separated on NuPAGE Novex 4%–12% gels (Invitrogen, Carlsbad, CA). Separated proteins were transferred to membranes (polyvinylidene fluoride) which were blocked and incubated with a primary antibody overnight at 4°C. After membranes were incubated with a secondary antibody linked to horseradish peroxidase, immunoblots were visualized using an enhanced chemiluminescence reagent. Optical density of immunoblots was measured using analysis software (NIH ImageJ). Values were normalized to β‐actin.

### 2.4 Immunoprecipitation

Immunoprecipitation was performed as described previously (Mao & Wang, 2016). Briefly, brain tissue was lysed in a RIPA buffer. Lysates were centrifuged at 800 g (10 min, 4°C). We then collected supernatants for immunoprecipitation. Supernatant proteins (300–500 μg) were incubated in a solution containing a mouse antibody against Fyn or Src (2–3 μg). Immunocomplexes were precipitated with 50% protein A/G agarose/sepharose bead slurry (GE Healthcare). Precipitated proteins were analyzed by immunoblots with a rabbit antibody indicated.

### 2.5 SFK kinase activity assay

A Takara Universal Tyrosine Kinase Assay Kit (Clontech Laboratory, Inc., Mountain View, CA) was used to assess SFK kinase activity as described previously (D. Z. Liu et al., 2014; Mao et al., 2018). Briefly, we used a mouse antibody against Fyn or Src to immunoprecipitate a protein of interest from striatal or mPFC homogenates. Immunopurified Fyn or Src proteins and ATP were added to microplate wells that were covered with an immobilized tyrosine kinase substrate poly (Glu‐Tyr) at 37°C for 30 min. Wells were washed and blocked. A horseradish peroxidase–conjugated anti‐phosphotyrosine antibody was added into wells, followed by measurement of phosphorylated substrates in absorbance at 450 nm.

### 2.6 Antibodies and pharmacological agents

Several primary antibodies were used in this study (Table 1). These include rabbit antibodies against Src (RRID:AB_2106047; Cell Signaling), Fyn (RRID:AB_631528; Santa Cruz Biotechnology, Santa Cruz, CA), phosphorylated Y416 (RRID:AB_10013641; pY416, Cell Signaling), or β‐actin (RRID:AB_476693; MilliporeSigma), or mouse antibodies against Src (RRID:AB_2106058; Cell Signaling), or Fyn (RRID:AB_627642; Santa Cruz). The anti‐pY416 antibody reacts with the Src family members when they are phosphorylated at the conserved Y416 site. Information on validation of antibodies is available from the companies. DPCPX was purchased from Tocris (Minneapolis, MN) and was dissolved in dimethyl sulphoxide and 0.1 M sodium hydroxide. Stock solutions were diluted in saline. The final concentration was 15% (v/v) for dimethyl sulphoxide and 8% (v/v) for sodium hydroxide (Uzbay et al., 2007). DPCPX was freshly prepared at the day of experiments.

**TABLE 1:** Primary Antibodies Used

Antigen	Description of Immunogen	Source, host species, catalog No., clone or lot No., RRID	Concentration used (μg/ml)	Primary antibody blocking solution
β‐Actin	C‐terminal actin fragment: Ser‐Gly‐Pro‐Ser‐Ile‐Val‐His‐Arg‐Lys‐Cys‐Phe	Sigma‐Aldrich (St. Louis, MO), rabbit polyclonal, A2066, AB_476693	0.2	3% Nonfat milk‐PBS 0.1% Tween‐20
Fyn	A peptide mapping at the N‐terminus of human Fyn	Santa Cruz Biotechnology (Santa Cruz, CA), rabbit polyclonal, sc‐16, AB_631528	1	3% Nonfat milk‐PBS 0.1% Tween‐20
Fyn	Amino acids 85‐206 of human Fyn	Santa Cruz Biotechnology, mouse monoclonal, sc‐434, AB_627642	1	3% Nonfat milk‐PBS 0.1% Tween‐20
Src	Recombinant fusion protein corresponding to residues 1‐110 of human Src	Cell Signaling Technology (Beverly, MA), rabbit monoclonal, 2123, AB_2106047	1	3% Nonfat milk‐PBS 0.1% Tween‐20
Src	Recombinant fusion protein corresponding to residues 1‐110 of human Src	Cell Signaling Technology, mouse monoclonal, 2110, AB_2106058	1	3% Nonfat milk‐PBS 0.1% Tween‐20
pY416	A synthetic phosphopeptide corresponding to residues surrounding Tyr416 of human Src	Cell Signaling Technology, rabbit polyclonal, 6943, AB_10013641	1	3% Nonfat milk‐PBS 0.1% Tween‐20

### 2.7 Statistics

After the normality of data was tested, we utilized GraphPad Prism 6 (RRID:SCR_002798; GraphPad software, La Jolla, CA) to analyze data. We used a two‐tailed unpaired Student's *t*‐test or one‐ or two‐way analysis of variance (ANOVA) with a multiple comparison post hoc test. The level of statistical significance was determined by a *P* value less than 0.05. Test for outliers was not performed. No rats were excluded from the analysis.

## 3 RESULTS

### 3.1 Effects of DPCPX on SFK Y416 phosphorylation

**FIGURE 1:**
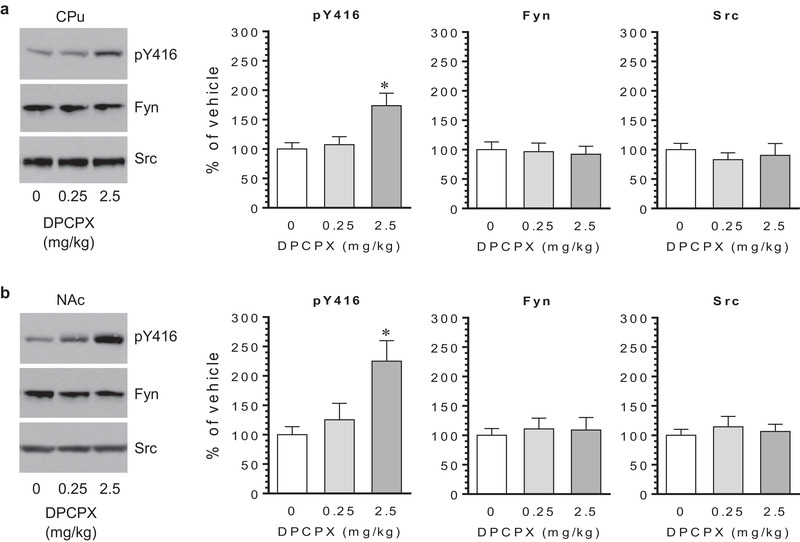
Effects of 8‐cyclopentyl‐1,3‐dipropylxanthine (DPCPX) on Src family kinase (SFK) Y416 phosphorylation in the rat striatum. (a) Effects of DPCPX on Y416 phosphorylation and expression of Fyn and Src in the caudate putamen (CPu). (b) Effects of DPCPX on Y416 phosphorylation and expression of Fyn and Src in the nucleus accumbens (NAc). Note that DPCPX at a higher rather than a lower dose significantly elevated pY416 levels in both the CPu and NAc, while DPCPX had no effect on total Fyn and Src expression. Representative immunoblots are shown to the left of the quantified data. Data are presented as means ± standard error of the mean (*n* = 4 per group) with ‘n’ equal to the number of rats and were analyzed with one‐way ANOVA: CPu/pY416: *F*_(2,9)_ = 6.400, *n* = 12, *p* = .019; CPu/Fyn: *F*_(2,9)_ = 0.080, *n* = 12, *p* = .923; CPu/Src: *F*_(2,9)_ = 0.317, *n* = 12, *p* = .736; NAc/pY416: *F*_(2,9)_ = 7.771, *n* = 12, *p* = .011; NAc/Fyn: *F*_(2,9)_ = 0.540, *n* = 12, *p* = .601; and NAc/Src: *F*_(2,9)_ = 0.283, *n *= 12, *p* = .760. *p < .05 versus vehicle.

We first examined whether blocking A_1_ receptors with DPCPX has any impact on SFK Y416 phosphorylation. To this end, we subjected rats to a single i.p. injection of vehicle or DPCPX at either 0.25 or 2.5 mg/kg. Rats were then sacrificed 20 min after DPCPX injection. Changes in Y416 phosphorylation were analyzed in different brain regions using western blots. We found that DPCPX at 0.25 mg/kg did not alter pY416 levels in the CPu (Figure 1A). Remarkably, DPCPX at 2.5 mg/kg markedly increased pY416 levels in the region. Similarly, DPCPX altered Y416 phosphorylation in the NAc. While DPCPX at 0.25 mg/kg had no effect, DPCPX at 2.5 mg/kg elevated pY416 levels in the NAc (Figure 1B). At either dose, DPCPX did not affect cellular levels of total Fyn and Src proteins in the CPu and NAc. These data indicate that pharmacological blockade of A_1_ receptors with a selective antagonist upregulates SFK Y416 phosphorylation in the CPu and NAc.

**FIGURE 2:**
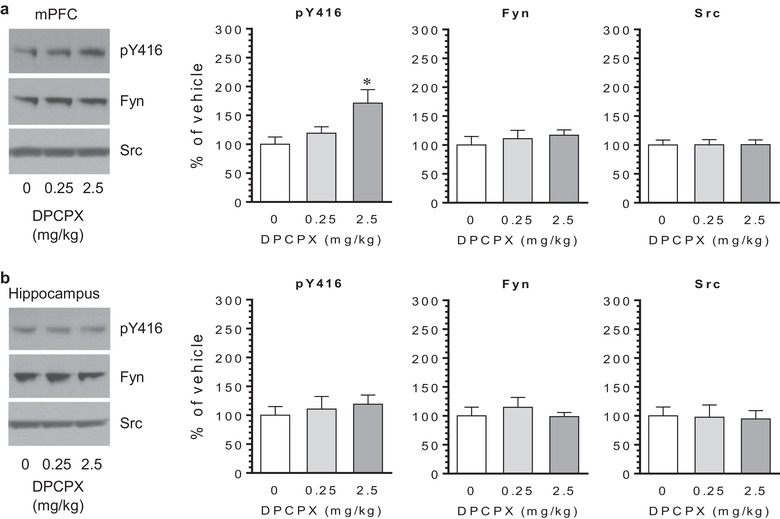
Effects of 8‐cyclopentyl‐1,3‐dipropylxanthine (DPCPX) on Src family kinase (SFK) Y416 phosphorylation in the rat medial prefrontal cortex (mPFC) and hippocampus. (a) Effects of DPCPX on Y416 phosphorylation and expression of Fyn and Src in the mPFC. (b) Effects of DPCPX on Y416 phosphorylation and expression of Fyn and Src in the hippocampus. Note that DPCPX at a higher rather than lower dose significantly elevated pY416 levels in the mPFC, while DPCPX had no effect on total Fyn and Src expression. No change in pY416 levels in the hippocampus was found following DPCPX administration. Representative immunoblots are shown to the left of the quantified data. Rats were given an i.p. injection of vehicle (0 mg/kg of DPCPX) or DPCPX (0.25 or 2.5 mg/kg) and were sacrificed 20 min after drug injection for immunoblot analysis. Data are presented as means ± standard error of the mean (*n* = 4 per group) with ‘n’ equal to the number of rats and were analyzed with one‐way ANOVA: mPFC/pY416: *F*_(2,9)_ = 4.884, *n* = 12, *p* = .036; mPFC/Fyn: *F*_(2,9)_ = 0.443, *n* = 12, *p* = .655; mPFC/Src: *F*_(2,9)_ = 0.003, *n* = 12, *p* = .998; hippocampus/pY416: *F*_(2,9)_ = 0.290, *n* = 12, *p* = .755; hippocampus/Fyn: *F*_(2,9)_ = 0.431, *n* = 12, *p* = .663; and hippocampus/Src: *F*_(2,9)_ = 0.024, *n* = 12, *p* = .977. *p < .05 versus vehicle.

In addition to the CPu and NAc, we assessed responses of SFK Y416 to DPCPX in the mPFC and hippocampus. As shown in Figure 2A, DPCPX was effective in the mPFC. The pY416 level in this region was elevated following DPCPX administration at a dose of 2.5 mg/kg. At 0.25 mg/kg, DPCPX induced a minimal change in pY416 levels. In the hippocampus, DPCPX failed to induce a significant change. The hippocampal pY416 level remained stable after an injection of DPCPX at either dose (Figure 2B). In both mPFC and hippocampus, DPCPX at two doses did not alter total Fyn and Src expression. Thus, the mPFC like the striatum is sensitive to DPCPX, while the hippocampus is not. Blocking A_1_ receptors leads to an increase in SFK Y416 phosphorylation in the mPFC.

### 3.2 Time‐dependent effects of DPCPX on SFK Y416 phosphorylation

**FIGURE 3:**
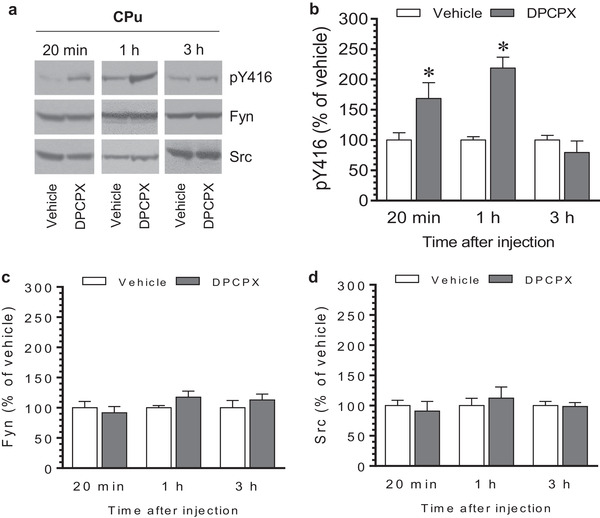
Time‐dependent effects of 8‐cyclopentyl‐1,3‐dipropylxanthine (DPCPX) on Src family kinase (SFK) Y416 phosphorylation in the rat caudate putamen (CPu). (a) Representative immunoblots illustrating time‐dependent effects of DPCPX on Y416 phosphorylation and expression of Fyn and Src in the CPu. (b–d) Quantifications of effects of DPCPX on Y416 phosphorylation (b) and expression of Fyn (c) and Src (d) in the CPu. Note that DPCPX induced a time‐dependent increase in pY416 levels, while DPCPX had no effect on total Fyn and Src expression. Rats were given an i.p. injection of vehicle or DPCPX (2.5 mg/kg) and were sacrificed at different time points (20 min, 1 h, and 3 h) after drug injection for immunoblot analysis. Data are presented as means ± standard error of the mean (*n* = 4–5 per group) with ‘n’ equal to the number of animals and were analyzed with unpaired Student's *t*‐test: pY416/20 min: *t*_(8)_ = 2.428, *n* = 10, *p* = .041; pY416/1 h: *t*_(6)_ = 6.286, *n* = 8, *p* < .001; pY416/3 h: *t*_(6)_ = 0.357, *n* = 8, *p* = .998; Fyn/20 min: *t*_(8)_ = 0.590, n = 10, *p* = .561; Fyn/1 h: *t*_(6)_ = 1.613, *n* = 8, *p* = .158; Fyn/3 h: *t*_(6)_ = 0.818, n = 8, *p* = .445; Src/20 min: *t*_(8)_ = 0.495, *n* = 10, *p* = .634; Src/1 h: *t*_(6)_ = .556, n = 8, *p* = .598; and Src/3 h: *t*_(6)_ = 0.162, *n* = 8, *p* = .876. *p < .05 versus vehicle at the same time points.

We next attempted to determine the temporal profile of the effect of DPCPX on SFK Y416 phosphorylation. Rats received an i.p. injection of vehicle or DPCPX (2.5 mg/kg) and were sacrificed at three different time points (20 min, 1 h, and 3 h) after drug injection. Changes in pY416 levels in three brain regions (CPu, NAc, and mPFC) were assayed using western blots. In the CPu, DPCPX induced a significant increase in pY416 levels at an early time point (20 min, Figure 3A and B), similar to the above finding. At 1 h after DPCPX injection, a marked increase in pY416 levels remained. However, an insignificant change in pY416 levels in the CPu was found at 3 h in DPCPX‐treated rats compared to vehicle‐treated rats. At all three time points surveyed, total Fyn and Src protein levels remained stable (Figure 3C for Fyn and Figure 3D for Src). These results indicate that DPCPX induced a rapid and reversible increase in SFK Y416 phosphorylation in the CPu.

**FIGURE 4:**
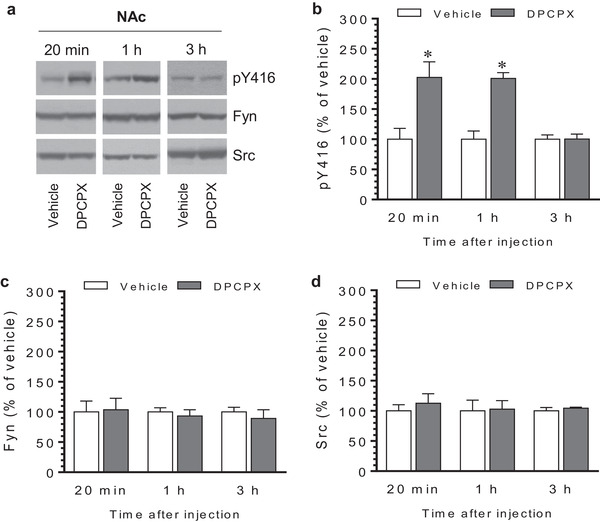
Time‐dependent effects of 8‐cyclopentyl‐1,3‐dipropylxanthine (DPCPX) on Src family kinase (SFK) Y416 phosphorylation in the rat nucleus accumbens (NAc). (a) Representative immunoblots illustrating time‐dependent effects of DPCPX on Y416 phosphorylation and expression of Fyn and Src in the NAc. (b–d) Quantifications of effects of DPCPX on Y416 phosphorylation (b) and expression of Fyn (c) and Src (d) in the NAc. Note that DPCPX induced a time‐dependent increase in pY416 levels, while DPCPX had no effect on total Fyn and Src expression. Rats were given an i.p. injection of vehicle or DPCPX (2.5 mg/kg) and were sacrificed at different time points (20 min, 1 h, and 3 h) after drug injection for immunoblot analysis. Data are presented as means ± standard error of the mean (*n* = 4–5 per group) with ‘n’ equal to the number of animals and were analyzed with unpaired Student's *t*‐test: pY416/20 min: *t*_(8)_ = 3.292, *n* = 10, *p* = .011; pY416/1 h: *t*_(6)_ = 3.828, n = 8, *p* = .009; pY416/3 h: *t*_(6)_ = 0.042, *n* = 8, *p* = .968; Fyn/20 min: *t*_(8)_ = 0.143, *n* = 10, *p* = .890; Fyn/1 h: *t*_(6)_ = 0.530, *n* = 8, *p* = .615; Fyn/3 h: *t*_(6)_ = 0.649, *n* = 8, *p* = .540; Src/20 min: *t*_(8)_ = 0.660, *n* = 10, *p* = .528; Src/1 h: *t*_(6)_ = 0.123, n = 8, *p* = .906; and Src/3 h: *t*_(6)_ = 0.793, *n* = 8, *p* = .458. *p < .05 versus vehicle at the same time points.

**FIGURE 5:**
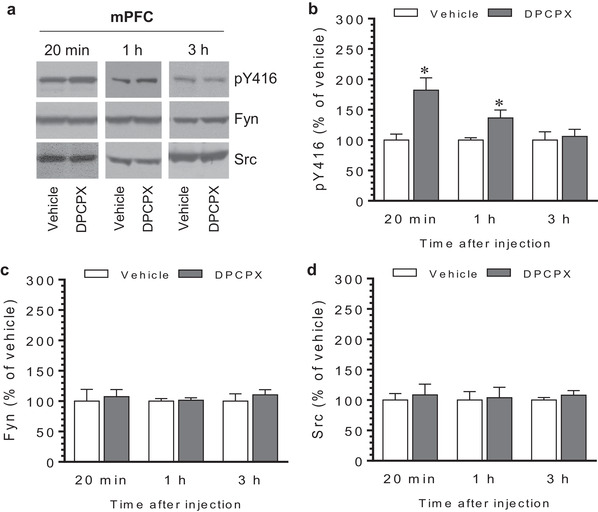
Time‐dependent effects of 8‐cyclopentyl‐1,3‐dipropylxanthine (DPCPX) on Src family kinase (SFK) Y416 phosphorylation in the rat medial prefrontal cortex (mPFC). (a) Representative immunoblots illustrating time‐dependent effects of DPCPX on Y416 phosphorylation and expression of Fyn and Src in the mPFC. (b–d) Quantifications of effects of DPCPX on Y416 phosphorylation (b) and expression of Fyn (c) and Src (d) in the mPFC. Note that DPCPX induced a time‐dependent increase in pY416 levels, while DPCPX had no effect on total Fyn and Src expression. Rats were given an i.p. injection of vehicle or DPCPX (2.5 mg/kg) and were sacrificed at different time points (20 min, 1 h, and 3 h) after drug injection for immunoblot analysis. Data are presented as means ± standard error of the mean (*n* = 4–5 per group) with ‘n’ equal to the number of animals and were analyzed with unpaired Student's *t*‐test: pY416/20 min: *t*_(8)_ = 3.593, *n *= 10, *p* = .01; pY416/1 h: *t*_(6)_ = 2.732, *n* = 8, *p* = .034; pY416/3 h: *t*_(6)_ = 0.335, *n* = 8, *p* = .749; Fyn/20 min: *t*_(8)_ = 0.329, *n* = 10, *p* = .751; Fyn/1 h: *t*_(6)_ = 0.278, *n* = 8, *p* = .790; Fyn/3 h: *t*_(6)_ = 0.707, *n* = 8, *p* = .506; Src/20 min: *t*_(8)_ = 0.418, *n* = 10, *p* = .687; Src/1 h: *t*_(6)_ = .179, *n* = 8, *p* = .864; and Src/3 h: *t*_(6)_ = 0.918, n = 8, *p* = .394. *p < .05 versus vehicle at the same time points.

Similar results were observed in the NAc. An increase in pY416 levels was seen in this region at 20 min and 1 h after DPCPX injection (Figure 4A and B). The increase declined to a level insignificantly different from the vehicle control at 3 h. No change in expression of total Fyn and Src in the NAc was seen at three time points (Figure 4C for Fyn and Figure 4D for Src). In the mPFC, we observed a marked increase in pY416 levels 20 min after DPCPX injection (Figure 5A and B). The increase persisted at 1 h in DPCPX‐treated rats compared to vehicle‐treated rats. At 3 h, pY416 levels were not significantly different between two groups. Fyn and Src expression was not altered following DPCPX administration (Figure 5C and D). Thus, like the CPu, the NAc and mPFC exhibit a similar dynamic pattern of SKF Y416 phosphorylation in response to DPCPX.

### 3.3 Effects of DPCPX on Y416 phosphorylation in immunopurified SFK members

**FIGURE 6:**
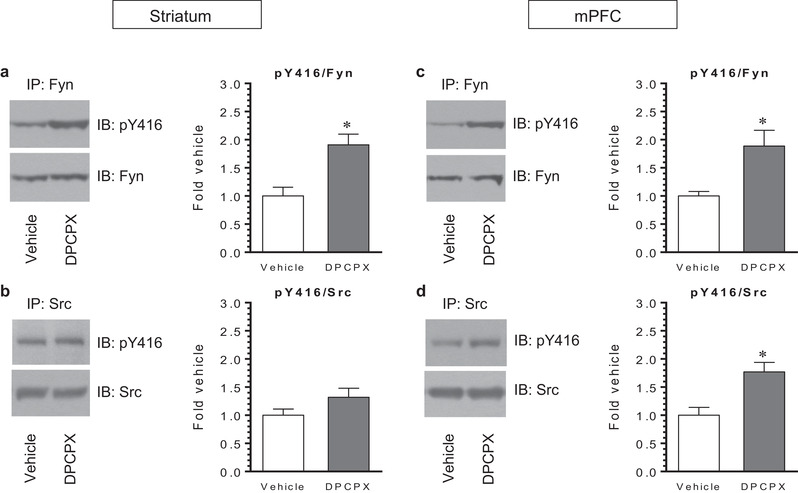
Effects of 8‐cyclopentyl‐1,3‐dipropylxanthine (DPCPX) on Y416 phosphorylation of immunopurified Src family kinase (SFK) members in the rat striatum and medial prefrontal cortex (mPFC). (a and b) Effects of DPCPX on Y416 phosphorylation of immunopurified Fyn (a) and Src (b) proteins in the striatum. (c and d) Effects of DPCPX on Y416 phosphorylation of immunopurified Fyn (c) and Src (d) proteins in the mPFC. Note that DPCPX enhanced pY416 levels in Fyn but not Src in the striatum and in both Fyn and Src in the mPFC. Representative immunoblots (IB) are shown to the left of the quantified data. Rats were given an i.p. injection of vehicle or DPCPX (2.5 mg/kg) and were sacrificed 20–30 min after drug injection. Fyn and Src proteins were purified from the striatum and mPFC via immunoprecipitation (IP) with a specific antibody indicated. Data are presented as means ± standard error of the mean (*n* = 4 per group) with ‘n’ equal to the number of animals and were analyzed with unpaired Student's *t*‐test: striatum (pY416/Fyn): *t*_(6)_ = 3.679, *n* = 8, *p* = .010; striatum (pY416/Src): *t*_(6)_ = 1.626, *n* = 8, *p* = .155; mPFC (pY416/Fyn): *t*_(6)_ = 3.016, *n* = 8, *p* = .023; and mPFC (pY416/Src): *t*_(6)_ = 3.553, *n* = 8, *p* = .012. *p < .05 versus vehicle.

To identify the member(s) of SFKs that respond to the A_1_ receptor antagonist, we combined western blots with immunoprecipitation. After an i.p. injection of DPCPX (2.5 mg/kg), rats were sacrificed 20–30 min after drug injection. Fyn and Src proteins were immunoprecipitated from striatal and mPFC lysates. Phosphorylation at Y416 was then analyzed in Fyn and Src immunoprecipitates using western blots. In the striatum containing the CPu and NAc, a significant increase in pY416 levels was shown in immunopurified Fyn proteins in DPCPX‐treated rats compared to vehicle‐treated rats (Figure 6A). The pY416 signal in Src immunoprecipitates showed a tendency of an increase after DPCPX administration, although it did not reach a statistically significant level (Figure 6B). In the mPFC, we observed a significant increase in pY416 levels in both Fyn and Src precipitates after DPCPX administration (Figure 6C and D). These results indicate that DPCPX upregulates Y416 phosphorylation of Fyn rather than Src proteins in the striatum, while DPCPX enhances Y416 phosphorylation of Fyn and Src in the mPFC.

### 3.4 Effects of DPCPX on Fyn and Src tyrosine kinase activity

**FIGURE 7:**
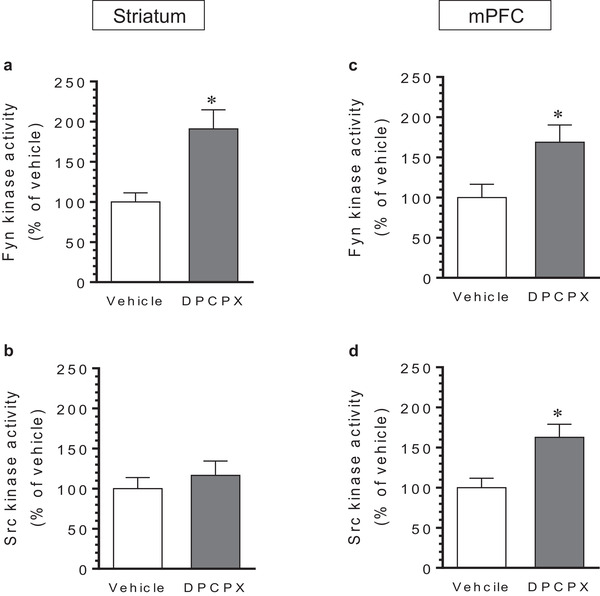
Effects of 8‐cyclopentyl‐1,3‐dipropylxanthine (DPCPX) on Fyn and Src kinase activity in the rat striatum and medial prefrontal cortex (mPFC). (a and b) Effects of DPCPX on Fyn (a) and Src (b) kinase activity in the striatum. (c and d) Effects of DPCPX on Fyn (c) and Src (d) kinase activity in the mPFC. Note that DPCPX increased Fyn but not Src kinase activity in the striatum and both Fyn and Src kinase activity in the mPFC. Rats were given an i.p. injection of vehicle or DPCPX (2.5 mg/kg) and were sacrificed 20–30 min after drug injection. Tyrosine kinase activity levels were measured in immunopurified Fyn and Src proteins from the striatum and mPFC. Data are presented as means ± standard error of the mean (*n* = 5 per group) with ‘n’ equal to the number of animals and were analyzed with unpaired Student's *t*‐test: striatum/Fyn: *t*_(8)_ = 3.473, *n* = 10, *p* = 0.008; striatum/Src: *t*_(8)_ = 0.722, *n* = 10, *p* = .491; mPFC/Fyn: *t*_(8)_ = 2.536, *n* = 10, *p* = .035; and mPFC/Src: *t*_(8)_ = 3.119, *n* = 10, *p* = .014. *p < .05 versus vehicle.

Given an elevated response of Y416 phosphorylation to DPCPX, SFK tyrosine kinase activity is reasoned to be increased by the antagonist. To determine this, rats were treated with DPCPX (2.5 mg/kg, i.p.; 20–30 min). They were then sacrificed and striatal and mPFC lysates were prepared. Fyn and Src proteins were immunoprecipitated from these lysates. Kinase activity levels of immunopurified Fyn and Src were assayed. As shown in Figure 7A, DPCPX elevated Fyn kinase activity in the striatum, while DPCPX did not significantly alter Src kinase activity in the region (Figure 7B). In the mPFC, DPCPX increased kinase activity of Fyn and Src precipitates in this region (Figure 7C and D). Thus, DPCPX possesses the ability to upregulate Fyn although not Src kinase activity in the striatum and both Fyn and Src kinase activity in the mPFC.

## 4 DISCUSSION

This study investigated the role of adenosine A_1_ receptors in the regulation of SFKs in the rat brain in vivo. We found that a systemic injection of the A_1_ antagonist DPCPX at a higher dose (2.5 mg/kg) but not a lower dose (0.25 mg/kg) induced a substantial increase in the autophosphorylation of SFKs at Y416 in the two subdivisions of the striatum (CPu and NAc). DPCPX also increased Y416 phosphorylation in the mPFC but not the hippocampus. The increase in Y416 phosphorylation was rapid and reversible as revealed in a time course study. The DPCPX‐elevated Y416 phosphorylation occurred in immunopurified Fyn but not Src proteins from the striatum. In the mPFC, both Fyn and Src precipitates showed an increase in Y416 phosphorylation in response to DPCPX. Finally, a similar pattern of responses of Fyn and Src in their tyrosine kinase activity was observed in the striatum and mPFC after DPCPX administration. Our results demonstrate a significant response of SFKs to changing A_1_ receptor signals. A_1_ receptors seemingly exert an inhibitory regulation of Fyn and/or Src phosphorylation and activity in the striatum and mPFC under normal conditions.

There exists a basal level of extracellular adenosine under normal conditions (Ballesteros‐Yanez et al., 2018). As a neuromodulator, adenosine through interacting with A_1_ receptors exerts a significant tonic modulation of functions of striatal neurons. For instance, an early study shows that a single dose of DPCPX and the nonselective adenosine receptor antagonist caffeine increased expression of the immediate early genes (c‐fos, zif/268, and arc) in the striatum at both mRNA and protein levels (Dassesse et al., 1999). DPCPX after an i.p. or intrastriatal injection enhanced damage to striatal neurons induced by a mitochondrial complex II inhibitor malonate (Alfinito et al., 2003). An A_1_ receptor agonist 2‐chloro‐N(6)‐cyclopentyladenosine significantly altered Thr34 phosphorylation of dopamine‐ and cAMP‐regulated phosphoprotein of 32 kDa (DARPP‐32) in the striatum (Yabuuchi et al., 2006), whereas A_2A_ receptors were also essential for the regulation of striatal DARPP‐32 phosphorylation (Shen et al., 2013). In addition, sleep deprivation decreased PKA phosphorylation in the striatum and hippocampus and impaired memory (Oliveira et al., 2019). DPCPX prevented the decrease in PKA phosphorylation in the striatum but not hippocampus and reversed the memory impairment. In this study, we found that the A_1_ antagonist DPCPX upregulated Fyn phosphorylation and activity in the striatum. These results are consistent with a general model that G_αi/o_‐coupled A_1_ receptors exert an inhibitory role in regulating multiple striatal activities, including the SFK/Fyn signaling pathway discovered in the present study. Of note, as compared to the striatum, SFKs in the hippocampus may be regulated by adenosine receptors differently. Postsynaptic A_2A_ receptors played a pivotal role in activating Src to promote long‐term potentiation of NMDA receptor‐mediated currents at hippocampal mossy fiber synapses (Rebola et al., 2008). In this study, blockade of A_1_ receptors had an insignificant impact on SFKs in Y416 phosphorylation in the hippocampus.

Precise mechanisms underlying the effect of DPCPX on Fyn in the striatum are unclear. Postsynaptically, A_1_ receptors are expressed in striatonigral projection neurons (Fuxe et al., 2007, 2010). Thus, the A_1_ receptor antagonist DPCPX is thought to mainly affect these neurons. Given the fact that A_1_ receptors are coupled to G_αi/o_ proteins, blockade of A_1_ receptors with DPCPX could result in the removal of the G_αi/o_‐mediated tonic inhibition of adenylyl cyclase, leading to an increase in cAMP production and PKA activity. The activated cAMP/PKA pathway could then elevate Fyn autophosphorylation in striatonigral neurons. Early studies have established a positive linkage from the cAMP/PKA pathway to SFK/Fyn activity (Schmitt & Stork, 2002 ; Yang et al., 2011; Yeo et al., 2011), which supports the notion that A_1_ receptors regulate Fyn activity via a signaling pathway involving cAMP and PKA. Alternatively, A_1_ receptors have been found to reside on incoming dopaminergic terminals within the striatum (Borycz et al., 2007). These presynaptic A_1_ heteroreceptors act to inhibit dopamine release (Borycz et al., 2007; O'Neill et al., 2007; Wood et al., 1989; Yabuuchi et al., 2006). Thus, DPCPX could block the presynaptic A_1_ receptor‐mediated inhibition of dopamine release and induce an increase in synaptic dopamine levels. Released dopamine could in turn activate D_1_ receptors specifically expressed in striatonigral neurons (Aubert et al., 2000; Bertran‐Gonzalez et al., 2010; Gerfen et al., 1990) to upregulate Fyn phosphorylation in these neurons. Previous work has consistently demonstrated phosphorylation of Fyn in the rat striatum in response to pharmacological activation of G_αs/olf_‐coupled D_1_ receptors (Mao & Wang, 2015 ; Mao et al., 2018). In addition to dopamine, presynaptic A_1_ heteroreceptors exert a tonic inhibition of glutamate release in the rat NAc (Quarta et al., 2004). Moreover, presynaptic A_1_ and A_2A_ receptors form heteromers in striatal glutamatergic nerve terminals (Ciruela et al., 2006; Ferré et al., 2007). This heteromerization provides a concentration‐dependent switch. Namely, low (under basal conditions) and high concentrations of adenosine preferentially activate A_1_ and A_2A_ receptors in the A_1_‐A_2A_ heteromer to inhibit and stimulate local glutamate release, respectively. It is likely that, in this study, DPCPX by blocking inhibitory A_1_ receptors could increase glutamate release to alter Fyn phosphorylation in the striatum. Future studies will have to elucidate precise pre‐ and/or postsynaptic mechanism(s) underlying the effect of DPCPX on Fyn phosphorylation in the striatum. Of note, A_1_ receptors are also expressed in peripheral tissues such as cardiac and vascular cells (Headrick et al., 2011). Further studies will clarify whether an A_1_ receptor agent injected i.p. could affect central Fyn phosphorylation indirectly via its effect on peripheral cardiovascular activities.

The expression level of Fyn is much greater than Src in the striatum (Pascoli et al., 2011). In the striatum, we found that DPCPX selectively affected Fyn. Unlike the striatum, DPCPX also increased phosphorylation of Src in addition to Fyn in the mPFC, indicating that A_1_ receptors are negatively coupled to Src in the mPFC. Of note, in contrast to this negative connection, A_1_ receptors could positively modulate Src in different models. For instance, an A_1_ receptor agonist activated Src to form a signaling pathway linking A_1_ receptors to activation of nuclear factor kappaB in HEK293 cells (A. M. F. Liu & Wong, 2004 ). An A_1_ receptor antagonist CPT reduced Src Y416 phosphorylation in acutely prepared mouse cortical slices containing somatosensory cortex (Deng et al., 2011).

Our studies on SFK phosphorylation were conducted in male rats. While sex differences in the role of A_1_ receptors in SFK phosphorylation are poorly understood at present, it is imperative to investigate the response of SFK phosphorylation to adenosine receptor agents in female animals in future studies.

## CONFLICT OF INTEREST

The authors declare that there is no potential conflict of interest.

### PEER REVIEW

The peer review history for this article is available at https://publons.com/publon/10.1002/brb3.2254.

## Data Availability

The data that support the findings of this study are available from the corresponding author upon reasonable request.
